# Ostéo arthrite tuberculeuse de la cheville et spondylodiscite: une association rare

**DOI:** 10.11604/pamj.2015.21.259.7326

**Published:** 2015-08-07

**Authors:** Samia Frioui, Sonia Jemni

**Affiliations:** 1 Service de Médecine Physique et de Réadaptation Fonctionnelle, CHU Sahloul, Faculté de Médecine Ibn El Jazzar, Sousse, Tunisie

**Keywords:** Tuberculose, spondylodiscite, cheville, arthrite, Tuberculosis, spondylodiscitis, ankle, arthritis

## Image en medicine

Une patiente âgée de 28 ans sans antécédents pathologiques et sans notion de contage tuberculeux, a été hospitalisée pour prise en charge rééducative d'une paraparésie post-spondylodiscite tuberculeuse multi-étagée découverte devant des dorsalgies de type mixte à irradiation intercostale avec fièvre, paresthésies, lourdeurs des deux membres inférieurs, fuites urinaires, le tout évoluant depuis 8 mois. La TDM et l'IRM rachidienne (A, B, C, D) ont montré: une collection épidurale en regard de D3D4D5D6, une destruction vertébrale de D4 avec recul du mur postérieur responsable d'une déformation dorsale avec compression médullaire, une atteinte D8D9D10D11D12 avec présence d'une collection intra-osseuse, une atteinte de L5 avec une petite collection vertébrale, une opacité alvéolaire de l'hémi-champs pulmonaire gauche. L'examen a trouvé un syndrome pyramidal prédominant à droite, une paraparésie et une douleur à la mobilisation de la cheville droite avec limitation articulaire. Les radiographies standards (E,F) étaient sans anomalies. Devant ce contexte, une ostéo-arthrite tuberculeuse de la cheville a été suspectée. La patiente a eu une arthrotomie montrant une hypertrophie synoviale pour laquelle elle a eu un lavage articulaire avec une synovectomie partielle. L'origine tuberculeuse a été confirmée par l'examen anatomo-pathologique. Concernant le rachis, la patiente a eu une immobilisation par un corset cervico-dorso-lombaire pendant 6 mois. L’évolution était marquée par la disparition des rachialgies, la récupération du déficit musculaire des deux membres inférieurs, la disparition des troubles vésico-sphinctériens, la disparition des douleurs et l'amélioration des amplitudes articulaires de la cheville droite.

**Figure 1:**
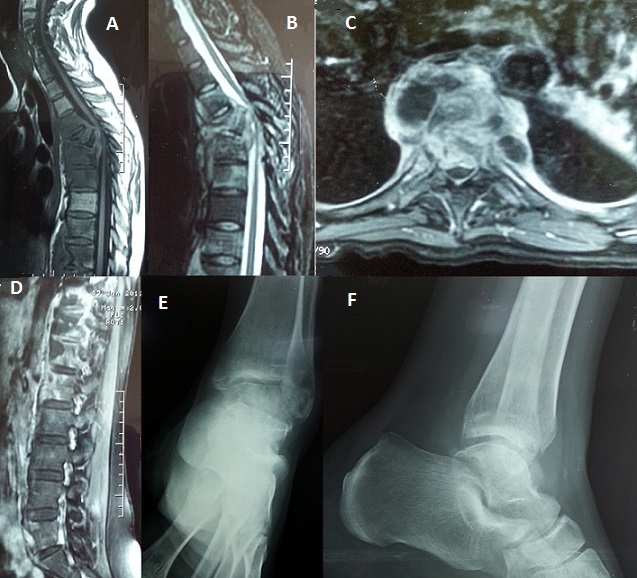
(A) IRM du rachis dorsal en coupe sagittale T1: destruction vertébrale de D4 avec recul du mur postérieur responsable d'une déformation dorsale avec compression médullaire; (B) IRM du rachis dorsal en coupe sagittale T2: destruction vertébrale de D4 avec recul du mur postérieur et compression médullaire, collection épidurale en regard de D3D4D5D6; (C) IRM médullaire en coupe transversale passant par D4: destruction vertébrale de D4 avec recul du mur postérieur; (D) IRM du rachis lombaire en coupe sagittale T2: atteinte de D10, D11, D12, L1 et L5 avec petite collection vertébrale; (E) radiographie de la cheville droite de face: absence de lyse osseuse; (F) radiographie de la cheville droite de profil: absence de signes en faveur d'une ostéo arthrite

